# Hydrogen sulphide suppresses human atrial fibroblast proliferation and transformation to myofibroblasts

**DOI:** 10.1111/jcmm.12114

**Published:** 2013-08-15

**Authors:** Jingwei Sheng, Winston Shim, Heming Wei, Sze Yun Lim, Reginald Liew, Tien Siang Lim, Boon Hean Ong, Yeow Leng Chua, Philip Wong

**Affiliations:** a Research and Development Unit, National Heart Centre SingaporeSingapore, Singapore; b Duke-NUS, Graduate Medical SchoolSingapore, Singapore; c Department of Cardiology, National Heart Centre SingaporeSingapore, Singapore; d Department of Cardiothoracic Surgery, National Heart Centre SingaporeSingapore, Singapore

**Keywords:** fibroblast, potassium channel, hydrogen sulphide, atrial fibrosis

## Abstract

Cardiac fibroblasts are crucial in pathophysiology of the myocardium whereby their aberrant proliferation has significant impact on cardiac function. Hydrogen sulphide (H_2_S) is a gaseous modulator of potassium channels on cardiomyocytes and has been reported to attenuate cardiac fibrosis. Yet, the mechanism of H_2_S in modulating proliferation of cardiac fibroblasts remains poorly understood. We hypothesized that H_2_S inhibits proliferative response of atrial fibroblasts through modulation of potassium channels. Biophysical property of potassium channels in human atrial fibroblasts was examined by whole-cell patch clamp technique and their cellular proliferation in response to H_2_S was assessed by BrdU assay. Large conductance Ca^2+^-activated K^+^ current (BK_Ca_), transient outward K^+^ current (I_to_) and inwardly rectifying K^+^ current (IK_ir_) were found in human atrial fibroblasts. Current density of BK_Ca_ (IC_50_ = 69.4 μM; *n* = 6), I_to_ (IC_50_ = 55.1 μM; *n* = 6) and IK_ir_ (IC_50_ = 78.9 μM; *n* = 6) was significantly decreased (*P* < 0.05) by acute exposure to NaHS (a H_2_S donor) in atrial fibroblasts. Furthermore, NaHS (100–500 μM) inhibited fibroblast proliferation induced by transforming growth factor-β1 (TGF-β1; 1 ng/ml), Ang II (100 nM) or 20% FBS. Pre-conditioning of fibroblasts with NaHS decreased basal expression of Kv4.3 (encode I_to_), but not KCa1.1 (encode BK_Ca_) and Kir2.1 (encode IK_ir_). Furthermore, H_2_S significantly attenuated TGF-β1–stimulated Kv4.3 and α-smooth muscle actin expression, which coincided with its inhibition of TGF-β–induced myofibroblast transformation. Our results show that H_2_S attenuates atrial fibroblast proliferation *via* suppression of K^+^ channel activity and moderates their differentiation towards myofibroblasts.

## Introduction

Cardiac fibroblasts are fundamentally involved in cardiac remodelling in normal ageing heart [1] and in damaged myocardium [2]. Aberrant proliferation of fibroblasts and their transformation to myofibroblasts is a hallmark of cardiac fibrosis, which is characterized by excessive extracellular matrix built-up leading to loss of tissue compliance [3, 4]. Because of their wide-ranging participation in myocardial pathophysiology, cardiac fibroblasts represent an attractive target in managing cardiac disorders, including cardiac hypertrophy, heart failure and arrhythmias [5]. Indeed, atrial fibrosis has been closely associated with atrial fibrillation [6, 7] and sinus node dysfunction [8].

Hydrogen sulphide (H_2_S) is an endogenously generated gaseous transmitter that has been reported to attenuate cardiac fibrosis [9]. It is known to mediate its effects by modulating ion channel activity in many cellular systems [10]. Hydrogen sulphide was the first opener of K_ATP_ channel identified in vascular smooth muscle cells [11]. Through activation of K_ATP_ channels, H_2_S lowers blood pressure, protects heart from ischaemia and reperfusion injury [12, 13]. We have recently reported that H_2_S inhibited delayed rectifier potassium channels in human iPS–derived cardiomyocytes [14]. Yet, effect of H_2_S on cardiac fibroblasts remains poorly understood. We hypothesized that H_2_S inhibits proliferation of atrial fibroblasts by inhibiting functioning of potassium channels. We present supporting data that H_2_S may potentially modulate cardiac fibrosis by inhibiting BK_Ca_, I_to_ and IK_ir_, independent of K_ATP_ channels, leading to decreased proliferation and suppression of transforming growth factor-β1 (TGF-β1)–induced myofibroblast transformation of atrial fibroblasts.

## Materials and methods

### Fibroblast isolation

Patients undergoing mitral valve repair and coronary bypass surgery (*n* = 10) were recruited after informed consent in protocol approved by institutional review board of Singapore General Hospital that conformed to the Declaration of Helsinki. Atrial appendages were collected as surgical by-product. Human atrial fibroblasts were isolated by mincing the appendages to less than 1 mm^3^ and followed by 0.1% trypsin digestion for 20 min. before plating onto tissue culture–treated 60-mm dishes to produce fibroblastic outgrowth from minced tissue pieces. The isolated fibroblasts were confirmed with expression of collagen I (1/20; Southern Biotech, Birmingham, AL, USA) and anti-human fibroblast (1/1000; Sigma-Aldrich, St. Louis, MO, USA) antibodies (Fig. S1). Atrial fibroblasts were passaged as monolayer in 10% foetal bovine serum–supplemented DMEM. Fibroblasts between passage 1 and 3 were used for subsequent experiments.

### Electrophysiological recordings

Cell were placed on the stage of a Nikon Diaphot inverted microscope and superfused continuously at 36 ± 1°C with Tyrode solution containing (in mM) 140 NaCl, 5.4 KCl, 1.8 CaCl_2_, 1 MgCl_2_, 10 HEPES and 10 Glucose (pH adjusted to 7.4 with NaOH). The patch-clamped cell was superfused by means of a temperature-controlled micro-superfusor (TC-324B, Warner Instruments, Hamden, CT, USA). Patch pipettes were made from borosilicate glass shanks (Sutter Instrument, Novato, CA, USA) and pulled with a Brown–Flaming puller (Model P-97; Sutter Instrument Co), and had tip resistances of 2–3 MΩ when filled with pipette solution. Pipette tips were polished (Microforge MF830; Narishige, Tokyo, Japan). These patch pipettes were filled with a standard solution containing (in mM) 140 KCl, 1.2 MgCl_2_, 0.05 EGTA, 10 HEPES, 0.1 GTP and 5.0 Mg ATP (pH adjusted to 7.2 with KOH). For Na^+^ current recording, the patch pipettes were filled with (in mM) 35 NaCl, 105 CsF, 0.1 EGTA and 10 HEPES (pH adjusted to 7.4 with CsOH). After a gigaohm seal was obtained by negative pressure suction, the cell membrane was ruptured by a gentle suction to establish whole-cell configuration with a seal resistance >800 MΩ. The cell membrane capacitance (40.27 ± 8.2 pF) was electrically compensated with the pulse software. The series resistance (Rs, 3–5 MΩ) was compensated by 50–70% to minimize voltage errors. Currents were elicited with voltage protocols as described in the following results section for different individual current recordings. Whole-cell voltage-clamp experiments were performed with an Axopatch 200B amplifier (Axon Instruments, Foster City, CA, USA) interfaced to a Digidata 1322A data acquisition system controlled by Clampex version 8.1 software (Axon Instruments). Data were analysed with pCLAMP software (Version 10.0; Axon Instrument) and Origin 8.0 (OriginLab, Northampton, MA, USA).

### Cell proliferation and apoptosis assay

Cell proliferation assay was performed with BrdU kit (Roche, Basel, Switzerland). Briefly, cells were plated on 96-well plate at a density of 3000/well and cultured for 24 hrs. After 4 hrs of serum starvation, cells were incubated for 24 hrs with medium containing ion channel blockers, NaHS or growth factors. BrdU labelling solution (100 μM) diluted 10 times in DMEM (0.1% FBS) was added to each well and the plates were incubated at 37°C for an additional 2 hrs. Incorporated BrdU was detected by an anti-BrdU antibody for 90 min. and colorimetric development proceeded for 15 min. before analysis by ELISA plate reader (SpectraMax, Molecular Device, Sunnyvale, CA, USA). Cellular apoptosis assay was performed with Caspase-3 Fluorescence Assay kit as instructed (Cayman Chemical, Ann Arbor, MI, USA). Briefly, cells were plated on 96-well plate at a density of 10^4^/well and cultured for 24 hrs. After 4 hrs of serum starvation, cells were incubated for 24 hrs with medium containing NaHS. Fluorescent intensity was obtained with ELISA plate reader (SpectraMax, Molecular Device) at 485 nm excitation and 535 nm emission wavelengths.

### RNA isolation and RT-PCR

Total RNA was extracted from human atrial fibroblasts with Trizol reagent (Life Technologies, Carlsbad, CA, USA) after 12 hrs of treatment. RT-PCR was performed with one-step kit (Invitrogen) where 1 μg RNA and random hexamer primer were used for the initiation of cDNA synthesis. Gene-specific primers for the BK_Ca_ (KCa1.1): forward 5′- GGAGGATGCCTCGAATATCA-3′; reverse 5′-AGCTCGGGATGTTTAGCAGA-3′; I_to_ (Kv4.3): forward 5′-CTGGACAAGAACCAGCGACAGTGCG-3′; reverse 5′-ATCACGATCAGGAGGGCCACATAGGG-3′ and IK_ir_ (Kir2.1): forward, 5′-TTGAGACCCAGACAACCATAGGCTATGG-3′; reverse 5′-TGGCCATGACTGCGCCAATGATG-3′; α-SMA: forward 5′-CATCACCAACTGGGACGACA-3′; reverse 5′-GTGGGTGACACCATCTCCAG- 3′; CSE: forward 5′-TCCGGATGGAGAAACACTTC-3′; reverse 5′-GCTGCCTTTAAAGCTTGACC-3′; K_ATP_ (Kir6.2): forward 5′-GACCCTCATCTTCAGCAAGC-3′; reverse 5′-GGTGTTGCCAAACTTGGAGT-3′; β-actin: forward 5′-TTTGAGACCTTCAACACCCC-3′; reverse 5′-TTTCGTGGATGCCACAGGA-3′. PCR products were fractionated on 2% agarose gel electrophoresis. Data were expressed as values of optical density (OD) standardized to those of β-actin.

### Immunocytochemistry

Atrial fibroblasts cultured on LabTek chamber slides (Nunc; Thermo Fisher Scientific, Waltham, MA, USA) were fixed with 4% paraformaldehyde, permeabilized with 0.5% Triton-X100 and blocked with 2% BSA. Cells were incubated overnight with antibodies against α-smooth muscle actin (1/2000; α-SMA; Sigma-Aldrich) to identify myofibroblasts, against anti-Kv1.1 (1/1000; Abcam, Cambridge, UK), anti-Kv4.3 (1/500; Abcam) and anti-Kir2.1 (1/1000; Abcam) to identify BK_Ca_, I_to_ and IK_ir_ channels, respectively (Fig. S2), before incubating with Alexa Fluor 488 or 555 secondary antibody (Life Technologies) and mounted in Vectashield mounting media containing DAPI for nuclear counterstain.

### Statistical analysis

Data were expressed as mean ± SE. Statistical significance of the difference between groups was determined with Student's *t*-test. A value of *P* < 0.05 was considered statistically significant.

## Results

### Hydrogen sulphide suppresses ion currents in human atrial fibroblasts

**Fig. 1:**
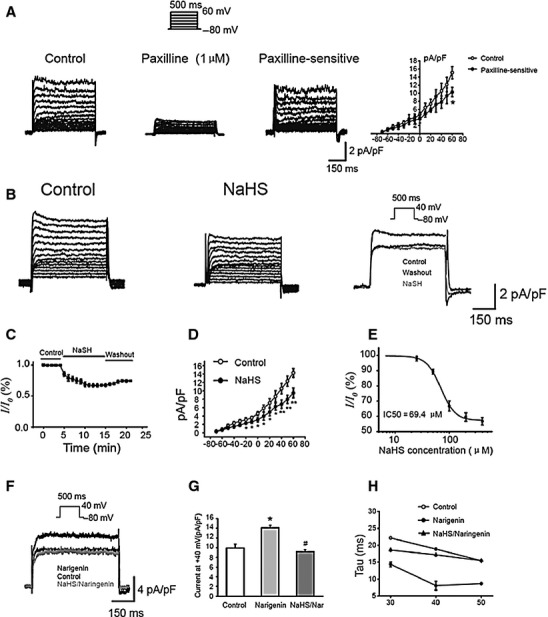
Effect of NaHS on BK_Ca_ currents in human atrial fibroblasts. (**A**) Voltage-dependent current was suppressed by BK_Ca_ blocker Paxilline (1 μM). Paxilline-sensitive I–V relationships of the membrane currents of typical BK_Ca_ channel. (**B**) BK_Ca_ traces recorded in the absence and presence of NaHS (100 μM). (**C**) Time course of BK_Ca_ current inhibition in human atrial fibroblast after addition of NaHS (100 μM). (**D**) Mean I–V relationship of peak BK_Ca_ current in the absence and presence of NaHS (100 μM) (**P < 0.01; *P < 0.05 *versus* control). (**E**) A concentration response curve of NaHS-induced inhibition on BK_Ca_. (**F**) Effect of NaHS (100 μM) on BK_Ca_ currents in the presence of Naringenin (10 μM). (**G**) Summarized data for peak BK_Ca_ currents at +40 mV at baseline, in the presence of Naringenin (10 μM), and in the presence of NaHS (100 μM) (*P < 0.05 *versus* basal levels; ^#^*P* < 0.05 *versus* Naringenin alone; *n* = 6). (**H**) Plot of the activation τ (τ_act_) as a function of membrane potential in the presence of Naringenin (10 μM) and Naringenin together with NaHS (100 μM) (**P < 0.01 *versus* basal levels; ^#^*P* < 0.05 *versus* Naringenin alone; *n* = 6).

Multiple ionic channels are reported to be expressed in human cardiac ventricular fibroblasts [15], ionic channels in our atrial fibroblasts were activated by depolarization voltage between −70 and +60 mV from a holding potential of −80 mV (0.2 Hz) to elicit total outward K^+^ currents. Activated currents that were sensitive to paxilline (1 mM), a specific BK_Ca_ inhibitor, were significantly suppressed at +60 mV, confirming the presence of BK_Ca_ current (52%; 163/309 cells) in human atrial fibroblasts (Fig. 1A). Under identical voltage-clamp condition, exposure to 100 μM NaHS (as a donor of H_2_S) similarly reduced the peak current density of BK_Ca_ (Fig. 1B). The inhibitory effects observed could not be washed out (Fig. 1C). The presence of NaHS resulted in a voltage-dependent suppression of the I–V curve from 10.5 ± 1.2 pA/pF to 6.8 ± 0.9 pA/pF at +40 mV (*P* < 0.01; *n* = 6) (Fig. 1D) and a dose-dependent inhibition of BK_Ca_ peak current density with an IC_50_ of 69.4 μM (Fig. 1E).

To verify the specificity of H_2_S inhibition on BK_Ca_, we assessed its effect in the presence of naringenin (10 μM), a specific opener of BK_Ca_ [16]. BK_Ca_ currents were elicited with clamp pulses at +40 mV from a holding potential of −80 mV under control condition (Fig. 1F). Compared with baseline (9.9 ± 0.8 pA/pF), naringenin increased BK_C_a current significantly (14.1 ± 0.5 pA/pF; *P* < 0.01; *n* = 6), but addition of NaHS returned naringenin-induced current to baseline (9.2 ± 0.4 pA/pF; *P* < 0.05; *n* = 6) (Fig. 1G). The rising phase of the BK_Ca_ currents at 50 mV with activation τ (τ_act_) at baseline (15.4 ± 0.1 ms) was lowered significantly by naringenin (8.6 ± 0.2 ms; *P* < 0.01; *n* = 6), but reversed to baseline after addition of NaHS (15.4 ± 0.2 ms; *P* < 0.05; *n* = 6), which confirmed its modulation of BK_Ca_ channel kinetics (Fig. 1H).

**Fig. 2:**
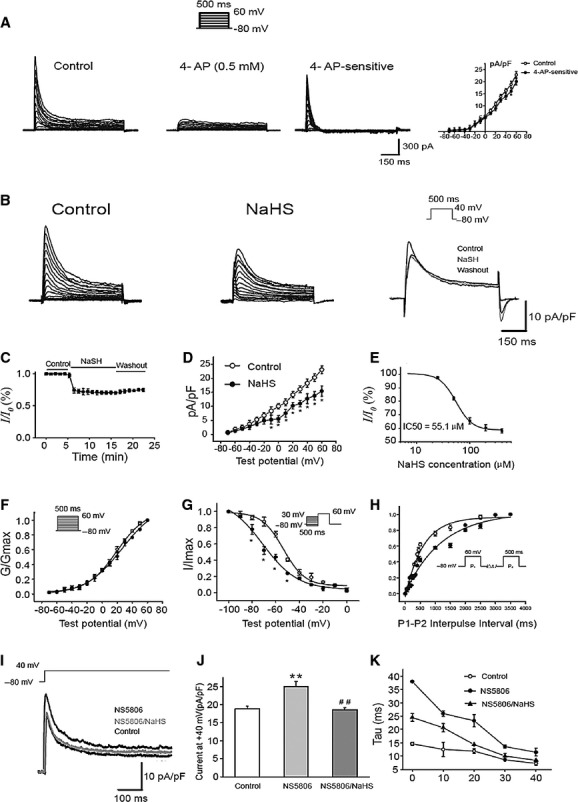
Effect of NaHS on I_to_ currents in human atrial fibroblasts. (**A**) Transient outward current was activated in traces recorded in the absence and presence of 4-AP (0.5 mM). 4-AP–sensitive I–V relationships of the membrane current of typical I_to_ channel. (**B**) I_to_ traces recorded in the absence and presence of NaHS (100 μM). (**C**) Time course of I_to_ current inhibition in human atrial fibroblast after addition of NaHS (100 μM). (**D**) Mean I–V relationship of peak I_to_ in the absence and presence of NaHS (100 μM) (*P < 0.05 *versus* control). (**E**) A concentration response curve of NaHS-induced inhibition on I_to_. (**F**) Mean voltage-dependent activation of I_to_ current and inactivation (**G**) and time-dependent recovery (**H**) in the absence and presence of NaHS (100 μM) (*P < 0.05 *versus* control). (**I**) Effect of NaHS (100 μM) on I_to_ currents in the presence of NS5806 (10 μM). (**J**) Summarized data for I_to_ at +40 mV at baseline, in the presence of NS5806 (10 μM), and in the presence of NS5806 together with NaHS (100 μM) (**P < 0.01 *versus* basal levels; ^##^*P* < 0.01 *versus* NS5806 alone; *n* = 6). (**K**) Mono-exponential functions were fitted to the current decays, and the time constants τ are shown as a function of membrane potential in the presence of NS5806 (10 μM) and in the presence of both NS5806 and NaHS (100 μM).

Similarly, under conditions to elicit total outward K^+^ currents, a 4-aminopyridine (4-AP; 0.5 mM)–sensitive current was detected, indicating the presence of transient outward currents, I_to_ (34%; 104/309 cells) in the atrial fibroblasts (Fig. 2A). Under identical voltage-clamp condition, exposure of fibroblasts to 100 μM NaHS reduced the peak current density of I_to_ (Fig. 2B). The inhibitory effects occurred within 1 min., reached saturation by 10 min. and could not be washed out (Fig. 2C). Addition of NaHS showed a voltage-dependent suppression of the I_to_ current in the I–V curve from 18.2 ± 1.5 pA/pF to 12.7 ± 1.7 pA/pF at +40 mV (*P* < 0.05; *n* = 6) (Fig. 2D) and demonstrated a dose-dependent inhibition of peak current density with an IC_50_ of 55.1 μM (Fig. 2E).

Steady-state activation of I_to_ was unaffected by NaHS (Fig. 2F). [The curves were fitted by the Boltzman equation: G/G_max_=1/[1 + exp(V_T_ − V_1/2_/κ)], where G/G_max_ represents a ratio of conductance to the maximum conductance, and V_T_ represents the values of the depolarizing pulses]. The half-maximum activation voltage (V_1/2_) and slope factor under control condition were 17.2 ±1.5 mV and 19.3 ± 1.3, respectively, which were not significantly different from those in the presence of NaHS (V_1/2_: 18.3 ± 1.2 mV, slope factor 20.2 ± 1.2) (*P* = NS; *n* = 6). In contrast, NaHS significantly influenced the steady-state inactivation of I_to_ (Fig. 2G). When fitted to Boltzman function, I/I_max_=1/[1 + exp(V_T_ − V_1/2_/κ)], the half-maximum inactivation voltage (V_1/2-inact_) and slope factor under control condition were −53.6 ± 1.2 mV and 9.08 ± 1.1, respectively, which were significantly different from those in the presence of NaHS (V_1/2-inact_: −71.1 ± 3.1 mV, slope factor 14.7 ± 2.4) (*P* < 0.05; *n* = 6). Furthermore, recovery of I_to_ from inactivation was analysed by delivering two identical 500 ms depolarizing pulses from −80 to +60 mV and varying the interpulse from 50 to 3500 ms. Addition of NaHS shifted the curve right and increased the half-recovery time of I_to_ from of 461.7 ± 57 to 1218.2 ± 49 ms. (*P* < 0.01; *n* = 6) (Fig. 2H), confirming inhibition of NaHS on the kinetic property of I_to_ channel recovery. Furthermore, these properties of I_to_ were similar to those reported in human ventricular fibroblasts [15].

The inhibitory effect of NaHS on I_to_ was further confirmed in the presence of NS5806 (10 μM), a specific opener of I_to_ [17] (Fig. 2I). The I_to_ currents were elicited with clamp pulses at +40 mV from a holding potential of −80 mV. Compared with baseline (18.8 ± 0.85 pA/pF), peak current density significantly increased (24.9 ± 1.5 pA/pF; *P* < 0.05; *n* = 6) after the addition of NS5806, but additional presence of NaHS (100 μM) returned the NS5806-stimulated currents to baseline levels (18.6 ± 0.6 pA/pF; *P* < 0.01; *n* = 6) (Fig. 2J). After exposure to NS5806 (10 μM), inactivation of I_to_ was significantly subdued, as reflected by an expansion in time constant (τ, from 8.6 ± 0.2 to 13.6 ± 0.7 ms at +30 mV, *P* < 0.05; *n* = 6). However, addition of 100 μM NaHS returned the time constant to 10.1 ± 0.9 ms at +30 mV in the presence of 10 μM NS5806 (Fig. 2K), confirming inhibition of H_2_S on I_to_ current.

**Fig. 3:**
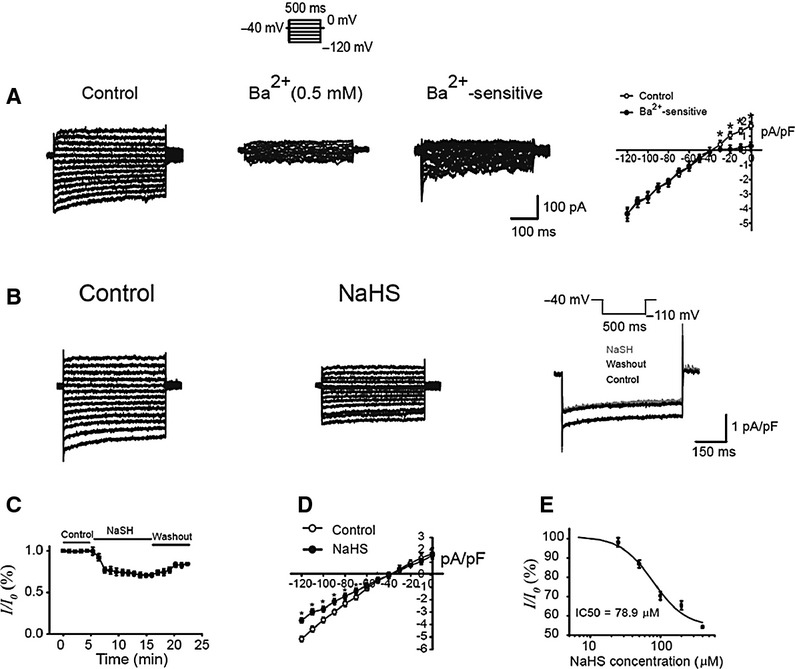
Effect of NaHS on IK_ir_ currents in human atrial fibroblasts. (**A**) inwardly rectifying voltage-dependent currents were suppressed by Ba^2+^ (0.5 mM). Ba^2+^-sensitive I–V relationships of the membrane currents of typical IK_ir_. (**B**) IK_ir_ traces recorded in the absence and presence of NaHS (100 μM). (**C**) Time course of IK_ir_ current inhibition after addition of NaHS (100 μM). (**D**) Mean I–V relationship of peak I_to_ current in the absence and presence of NaHS (100 μM) (*P < 0.05 *versus* control). (**E**) A concentration response curve of NaHS-induced inhibition on IK_ir_ (*P < 0.05; **P < 0.01; *n* = 6).

Besides BK_Ca_ and I_to_ currents, an inward rectifier current activated by hyperpolarization voltage steps on a holding potential of −40 mV that was sensitive to Ba^2+^ (0.5 mM) was found, indicating the presence of IK_ir_ inward current (28%; 28/309 cells) in the atrial fibroblasts (Fig. 3A). Exposure of atrial fibroblasts to 100 μM NaHS reduced the peak current density of IK_ir_ (Fig. 3B). The inhibitory effects occurred within 1 min., reached saturation at 10 min. and could not be washed out (Fig. 3C). NaHS showed a voltage-dependent suppression of the IK_ir_ current on the I–V curve from −4.4 ± 0.1 pA/pF to −3.0 ± 0.1 pA/pF at −110 mV (*P* < 0.05; *n* = 6) (Fig. 3D) and a dose-dependent inhibition of peak current density with an IC_50_ of 78.9 μM (Fig. 3E).

A minority of the atrial fibroblasts (1%; 1/54 cells) were found to exhibit inward currents with 50 ms voltage steps between −60 and +70 mV from −80 mV holding potential in 10 mV increments that resembled sodium current, indicating that K^+^ currents represent the major ionic species in human atrial fibroblasts.

### H_2_S inhibits proliferation of atrial fibroblasts *via* suppression of I_to_ currents and gene expression

**Fig. 4:**
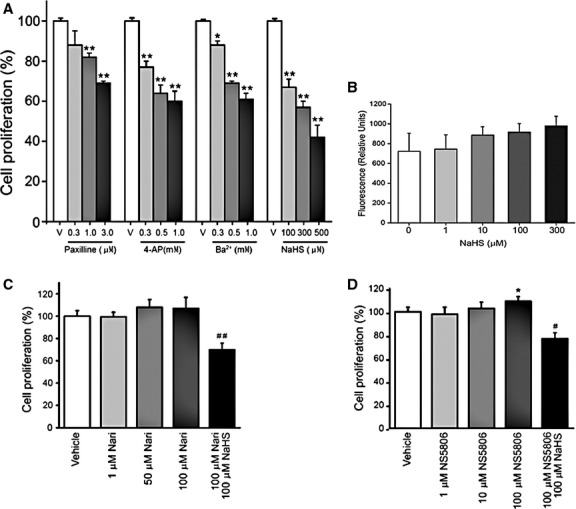
Effect of ion channel modulators on cell proliferation and apoptosis of human atrial fibroblasts. (**A**) Cell proliferation was assessed by BrdU assay in cells treated with Paxilline (0.3–3 μM), 4-AP (0.3–1 mM), Ba^2+^ (0.3–1 mM) or NaHS (100–500 μM) (*P < 0.05; **P < 0.01 *versus* basal levels; *n* = 10). (**B**) NaHS (1–300 μM) exerts no significant cellular apoptosis effect on cultured human atrial fibroblasts. (**C**) NaHS reverses fibroblast proliferation induced by Naringenin (Nari; BK_Ca_ opener, ^#^*P* < 0.05 *versus* Nari alone). (**D**) NaHS suppresses cellular proliferation induced by NS5806 (I_to_ opener, *P < 0.05 *versus* basal levels; ^##^*P* < 0.01 *versus* NS5806 alone; *n* = 10).

Inhibition of BK_Ca_ channel by paxilline, but not Na channel, has been reported to suppress proliferation of ventricular fibroblasts previously [18]. We investigated whether inhibition of the major K^+^ currents of BK_Ca_ and I_to_ by H_2_S similarly affected atrial fibroblast proliferation. Cell proliferation was found to be dose-dependently suppressed by paxilline (BK_Ca_ inhibitor), 4-AP (I_to_ inhibitor) and Ba^2+^ (IK_ir_ inhibitor) (Fig. 4). Similarly, NaHS at 100, 300, 500 μM reduced cell proliferation by 33.1 ± 4.2%, 43.7 ± 3.1%, 58.4 ± 6.2%, respectively (*P < 0.05; **P < 0.01 *versus* vehicle control; *n* = 10) without significant apoptotic effect observed at 300 μM (Fig. 4B). While naringenin (100 μM) had no effect on cellular proliferation, NS5806 (100 μM) enhanced fibroblast proliferation by 9.1 ± 5.0% (*P* < 0.05; *n* = 10). However, NaHS (100 μM) reduced cellular proliferation by 29.1 ± 5.8% (*P* < 0.01; *n* = 10) and 23.1 ± 4.8% (*P* < 0.05; *n* = 10) in the presence of naringenin (100 μM) and NS5806 (100 μM), respectively, confirming additive inhibitory effects of H_2_S on BK_Ca_ and I_to_ currents in reducing cellular proliferation (Fig. 4C and D).

**Fig. 5:**
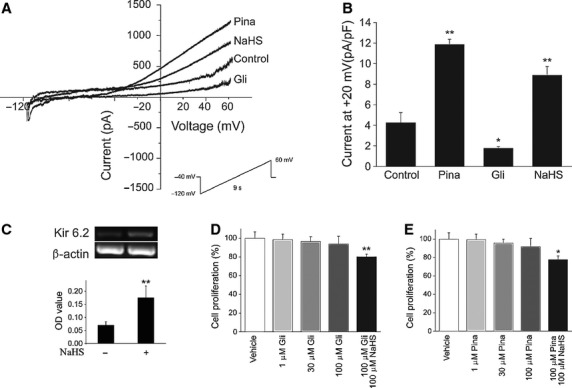
Effect of NaHS on K_ATP_ channels. (**A**) Superimposed K_ATP_ current traces recorded in the absence and presence of NaHS (100 μM), pinacidil (30 μM) and glibenclamide (100 μM) (*n* = 6 in each group). (**B**) Graph representation of mean values of K_ATP_ current in the absence and presence of NaHS (100 μM), pinacidil (30 μM) and glibenclamide (100 μM) (*P < 0.05; **P < 0.01 *versus* basal levels). (**C**) RT-PCR micrographs showing effect of 100 μM NaHS on Kir6.2 expression in atrial fibroblasts. Summary data displaying effect of NaHS on Kir6.2 expression. (**P < 0.01 *versus* basal levels; *n* = 4). (**D** and **E**) Cell proliferation was assessed in cells treated with glibenclamide (1–100 μM), pinacidil (1–100 μM) in the absence and presence of NaHS (100 μM). (*P < 0.05; **P < 0.01 *versus* basal levels; *n* = 4).

K_ATP_ channel has been reported to affect cellular proliferation [19]. However, modulation of K_ATP_ channel (30%; 22/73 cells) (Fig. 5A and B) and Kir6.2 (responsible for K_ATP_) gene expression (Fig. 5C) by H_2_S while confirming its role in enhancing current density, failed to show any appreciable effect on proliferation of our atrial fibroblasts. The K_ATP_ currents were elicited from voltage-clamped at the holding potential of −40 mV, voltage ramps were applied every 9 sec. from −120 mV to +60 mV at 20 mV/sec. and subsequently ramps to −40 mV at −100 mV/sec. Consistently, activation of the K_ATP_ channel by 30 μM pinacidil (specific channel enhancer) or its inhibition by 100 μM glibenclamide (specific channel inhibitor) did not significantly affect cellular proliferation despite the observed drastic modulation of current density (Fig. 5D and E). Down-regulation of fibroblast growth was observed only in the additional presence of NaHS with glibenclamide (19.9 ± 2.9% reduction *versus* control; *P* < 0.01; *n* = 4) or NaHS with pinacidil (22.5 ± 4.2% reduction *versus* control; *P* < 0.05; *n* = 4), suggesting that H_2_S inhibition of proliferation was independent of its modulating role of K_ATP_ channel in atrial fibroblasts.

**Fig. 6:**
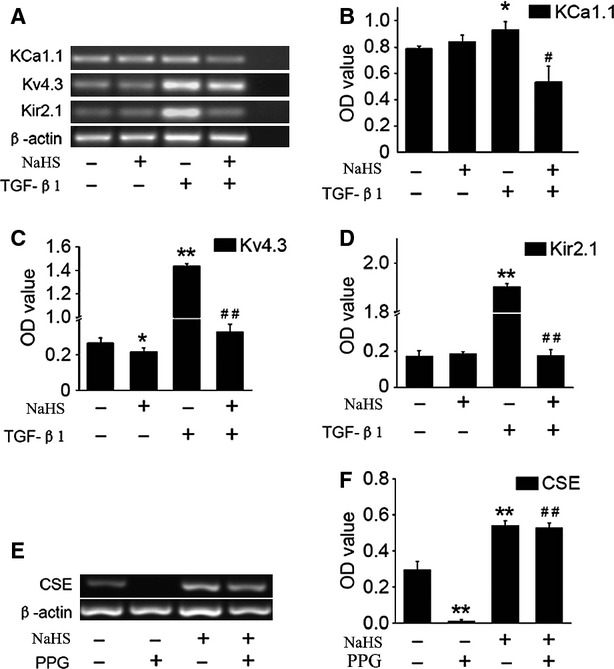
Effect of NaHS on ion channel and CSE expression. (**A**) RT-PCR micrographs of Kca1.1 (BK_Ca_), Kv4.3 (I_to_) and Kir2.1(Ik_ir_) expression in response to NaHS and transforming growth factor-β1 (TGF-β1). (**B**–**D**) Relative OD of PCR products. Each OD value is standardized to that of β-actin (*P < 0.05; **P < 0.01 *versus* basal levels; ^#^*P* < 0.05; ^##^*P* < 0.01 *versus* TGF-β1 alone; *n* = 4). (**E**) RT-PCR micrograph showing the expression of CSE in response to PPG (3 mM) and NaHS (100 μM). (**F**) Summary data displaying effect of NaHS on CSE expression in the absence and presence of 3 mM PPG (**P < 0.01 *versus* basal levels; ^##^*P* < 0.05 *versus* PPG alone; *n* = 5).

Gene expression showed that H_2_S reduced the mRNA level of KCa1.1 (responsible for BK_Ca_), Kv4.3 (responsible for I_to_), Kir2.1 (responsible for IK_ir_) in TGF-β1–stimulated fibroblasts by 42.3 ± 5.1% (*P* < 0.05; *n* = 4), 76.9 ± 3.5% (*P* < 0.01; *n* = 4), 90.8 ± 4.7% (*P* < 0.01; *n* = 4), respectively, at 12 hrs after addition of NaHS (Fig. 6A–D). Furthermore, pre-treatment with NaHS decreased mRNA level of Kv4.3 by 21.6 ± 2.2% (*n* = 4; *P* < 0.05 *versus* basal levels), but not that of KCa1.1 and Kir2.1. Furthermore, NaHS enhanced production of endogenous H_2_S by enhancing cystathionine γ–lyase (CSE) mRNA levels and maintaining its expression even in the presence of D,L-propargylgylcine (PPG), a potent inhibitor of CSE (Fig. 6E and F). These results indicated that H_2_S inhibited fibroblast proliferation by regulating Kv4.3 mRNA expression and inhibiting I_to_ current, possibly *via* an autocrine feedback mechanism.

### H_2_S inhibits TGF-β1–induced differentiation of atrial fibroblasts to myofibroblasts

**Fig. 7:**
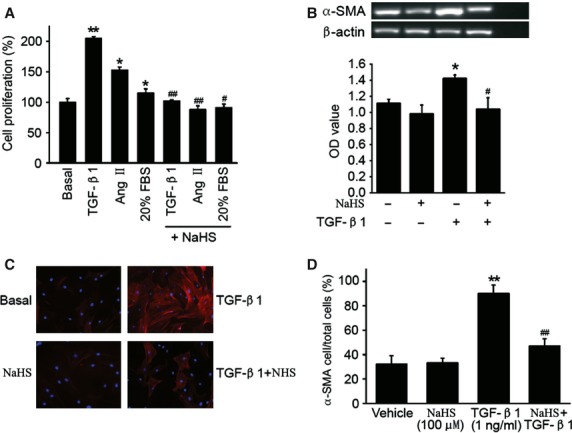
H_2_S donor inhibits cytokine-induced fibroblast proliferation and transforming growth factor-β1 (TGF-β1)–mediated myofibroblast transformation. (**A**) Proliferation of atrial fibroblasts in response to TGF-β1 (1 ng/ml), Ang II (100 nM) and 20% FBS in the absence and presence of NaHS (100 μM) (*P < 0.05; **P < 0.01 *versus* basal levels; ^#^*P* < 0.05; ^##^*P* < 0.01 *versus* TGF-β1, Ang II and 20% FBS alone; *n* = 10). (**B**) RT-PCR micrograph showing the effect of TGF-β1 on α-smooth muscle actin (α-SMA) expression in atrial fibroblasts with and without 100 μM NaHS pre-treatment. Summary data displaying NaHS inhibition of TGF-β1–induced α-SMA expression (*P < 0.05 *versus* basal levels; ^#^*P* < 0.05 *versus* TGF-β1 alone; *n* = 5). (**C**) Immunocytochemical staining of fibroblasts against α-SMA. Slides were counterstained with DAPI to visualize nuclei. Treatment of fibroblasts with 1 ng/ml TGF-β1 for 48 hrs induced a significant increase in expression of α-SMA that was attenuated by pre-treating fibroblasts with 100 μM NaHS for 48 hrs. Immunocytochemical data representative of four experiments in cells isolated from separate patient samples (*n* = 4). (**D**) Bar Graph representation of α-SMA–stained human fibroblasts. Cells were counted in 4 slides per group in 4 experiments (**P < 0.01 *versus* vehicle control; ^##^*P* < 0.01 *versus* TGF-β1 alone).

Transforming growth factor-β1 and Angiotensin II (Ang II) as the major mediators of fibroblast proliferation and their differentiation towards myofibroblasts in atrial fibrosis [20, 21] were consistently shown to promote proliferation of atrial fibroblasts in our study (Fig. 7A). Additional presence of NaHS decreased TGF-β1– (1 ng/ml), Ang II- (100 nM) and 20% FBS-induced fibroblast proliferation by 50.1 ± 4.3% (*P* < 0.01; *n* = 10), 42.1 ± 5.7% (*P* < 0.01; *n* = 10) and 21.2 ± 3.4% (*P* < 0.05; *n* = 10), respectively, which suggested H_2_S as a potent inhibitor of cytokine-mediated fibroblast proliferation. Furthermore, NaHS (100 μM) decreased TGF-β1-(1 ng/ml)–induced fibroblast transformation into myofibroblasts whereby mRNA expression of α-SMA, a hallmark of fibroblast differentiation, was significantly down-regulated (34.1 ± 7.1% reduction *versus* TGF-β1 alone; *P* < 0.05) (Fig. 7B), which was confirmed by reduced immunocytochemical α-SMA staining (percentage of α-SMA–positive cells, 47 ± 6% *versus* 90 ± 7%; *P* < 0.01; *n* = 4) (Fig. 7C and D). Nevertheless, no significant change in α-SMA–containing stress fibres was observed after NaHS treatment alone (percentage of α-SMA–positive cells, 33 ± 4%; *n* = 4) as compared with standard cultured atrial fibroblasts (32 ± 7%; *n* = 4) in 10% FBS.

## Discussion

Multiple potassium channels are known to express in cardiac ventricular fibroblasts [15] and inhibition of BK_Ca_ current resulted in suppression of fibroblast proliferation [18]. Transient outward K^+^ current, I_to_, is present in neonatal rat cardiac fibroblasts (encoded by Kv1.4) [22] and human ventricular fibroblasts (encoded by Kv4.3) [15]. Similarly, Ba^2+^-sensitive inward rectifier K^+^ current (encoded by Kir2.1/Kir2.3) is present in human ventricular fibroblasts [15] and rat ventricular fibroblasts [23] whereby its modulation may have major significance in cardiac fibrosis. However, their roles in atrial fibroblasts which are more actively participating in cardiac fibrosis [24], are relatively not well understood.

We demonstrated that H_2_S dose-dependently inhibited BK_Ca_, I_to_ and IK_ir_ in human atrial fibroblasts within minutes, suggesting an acute modulation of H_2_S on such channels. The inhibitory effect of H_2_S on BK_Ca_, I_to_ and IK_ir_ was observed at 25–400 μM. The physiological levels of plasma H_2_S have been reported to be 50–160 μM in human brain [25] and 50–100 μM in human serum [26]. As NaHS dissolved in saline, one-third of the H_2_S exists as an undissociated gas, and the remaining two-third as the HS^−^ anion [27]. Therefore, the physiologically relevant concentration of H_2_S (25–400 μM) used in this study, which effectively blocked BK_Ca_, I_to_ and IK_ir_ *in vitro*, is likely to be attainable *in vivo*.

We found that NaHS attenuated naringenin-induced BK_Ca_ activation and decelerated the transition from closed to open state of the channel, suggesting a role for H_2_S in regulating BK_Ca_ channel kinetic and voltage sensitivity. However, NaHS had no effect on the half-maximum voltage activation, but shifted the steady-state inactivation curve to the left, indicating that the voltage-dependent steady-state inactivation kinetics of I_to_ channel were altered. Furthermore, NaHS markedly shifted the recovery curve of I_to_ to the right, indicating that H_2_S attenuated the recovery of I_to_ from inactivation. These results indicated that H_2_S inhibited I_to_ through facilitation of steady-state inactivation and attenuation of recovery from inactivation. In contrast to reported presence of sodium current in ventricular fibroblasts (61%) [15], we found relatively few cells (1%) with detectable sodium current. This is consistent with previous reported presence of fast sodium current only in atrial myofibroblasts, but not in undifferentiated fibroblasts [28] like those used in our study.

BK_Ca_ channels (encoded by KCa1.1) have been demonstrated to regulate proliferation of human cardiac ventricular fibroblasts [18] and endothelial cells [29]. Furthermore, inhibition of IK_ir_ current suppressed proliferation of endothelial cells [30]. Similarly, inhibition of BK_Ca_ (by paxilline), I_to_ (by 4-AP) and IK_ir_ (by Ba^2+^) currents resulted in a significant reduction in fibroblast proliferation in our study. Consistently, suppression of the K^+^ currents by NaHS inhibited atrial fibroblast proliferation in a dose-dependent manner. Furthermore, suppression of proliferation by NaHS in the presence of naringenin (channel opener of BK_Ca_) or NS5806 (channel opener of I_to_) suggested an additive inhibitory effect of H_2_S on BK_Ca_ and I_to_ channels in proliferation of atrial fibroblasts. Consistent with K_ATP_ channel–activating effect of H_2_S [11, 31], addition of NaHS recovered K_ATP_ channel activity from glibenclamide inhibition. Nevertheless, suppression of cellular proliferation by NaHS in the presence of glibenclamide (specific inhibitor of K_ATP_) or pinacidil (specific enhancer of K_ATP_) indicated that K_ATP_ channel was unlikely to be involved in proliferation of atrial fibroblasts. Consistently, H_2_S inhibition of lung fibroblast proliferation has been reported to be independent of K_ATP_ channel [32].

Consistent with electrophysiological findings on the presence of BK_Ca_, I_to_ and IK_ir_ potassium currents, RT-PCR confirmed expression of KCa1.1, Kv4.3 and Kir2.1 in atrial fibroblasts. Furthermore, H_2_S decreased Kv4.3 expression and significantly moderated TGF-β1–mediated enhanced expression of Kv4.3 as well as KCa1.1 and Kir2.1. Effect of NaHS (exogenous donor of H_2_S) on expression of cystathionine γ-lyase (CSE) that produces endogenous H_2_S is controversial, with reports of no effect in human airway smooth muscle cells [33] to inhibitory effect in mouse aortic smooth muscle cells [34]. However, in concordance with other reports [27, 35], our results showed that NaHS enhanced CSE expression and further sustained its expression in the presence of DL-PPG[27] that strongly inhibited expression of CSE.

Myofibroblasts characterized by increased α-SMA expression are abundant in cardiac fibrosis [36] that has been associated with TGF-β-mediated [20] and Ang II-mediated [21] atrial fibrillation. Preventing myofibroblast differentiation from proliferating fibroblasts has been an attractive target in limiting cardiac fibrosis. Inhibition of TGF-β1 function by anti–TGF-β1 antibodies reduced myofibroblasts and lessened fibrosis [37]. Hydrogen sulphide was found to inhibit TGF-β–induced transformation of MRC5 lung fibroblasts to myofibroblasts [32]. Consistently, our results showed that NaHS effectively reduced proliferation of atrial fibroblasts in response to TGF-β1, Ang II or FBS. Furthermore, NaHS ameliorated transformation towards myofibroblasts whereby α-SMA expression and their stress fibres were significantly suppressed, although causal role of potassium channels in such transformation remained to be ascertained.

In summary, our study provides evidence of major K^+^ channels in human atrial fibroblasts that share similar heterogenous expression as in human ventricular fibroblasts [15]. Hydrogen sulphide inhibits fibroblast proliferation probably through a combined modulation of BK_Ca_, I_to_, IK_ir_, but not K_ATP_, channels. Although roles of MAPK and ERK pathways in our atrial fibroblasts remain to be determined, they were implicated in H_2_S-mediated suppression of proliferation of vascular smooth muscle cells [38] and lung fibroblasts [32]. Both kinase pathways were linked to cell cycle progression in lung fibroblasts [39], which, in turn were reportedly regulated by Bk_Ca_ in human ventricular fibroblasts [18]. However, K_ATP_ was found to play no significant role in ERK-inhibiting effect of H_2_S [32], which may explain our observation in this study. Consistent with the observed beneficial effects of H_2_S on cardiac fibrosis *in vivo* [12, 13], our results suggested that such effects may be partly mediated *via* selective inhibition of K^+^ channels in atrial fibroblasts and suppression of their transformation to myofibroblasts. Such regulating role of H_2_S in atrial fibroblasts may have clinical value in targeting atrial fibrillation, which invariably linked to atrial fibrosis.

## Supporting information

Figure S1 Immunocytochemical staining against anti-collagen I (top panel) and anti-human fibroblast antibodies in human atrial fibroblasts. Scale bar: 25 μm

Figure S2 Immunocytochemical staining of Bkca (Kv1.1), Ito (Kv4.3) and IKir (Kir2.1) channels in human atrial fibroblasts. Scale bar: 50 μm.
