# Supplementary figures and images for: Hydrogen sulphide suppresses human atrial fibroblast proliferation and transformation to myofibroblasts
Source: J Cell Mol Med. 2013 Aug 15;17(10):1345–54. doi: 10.1111/jcmm.12114 (PMC4159014; doi:10.1111/jcmm.12114)

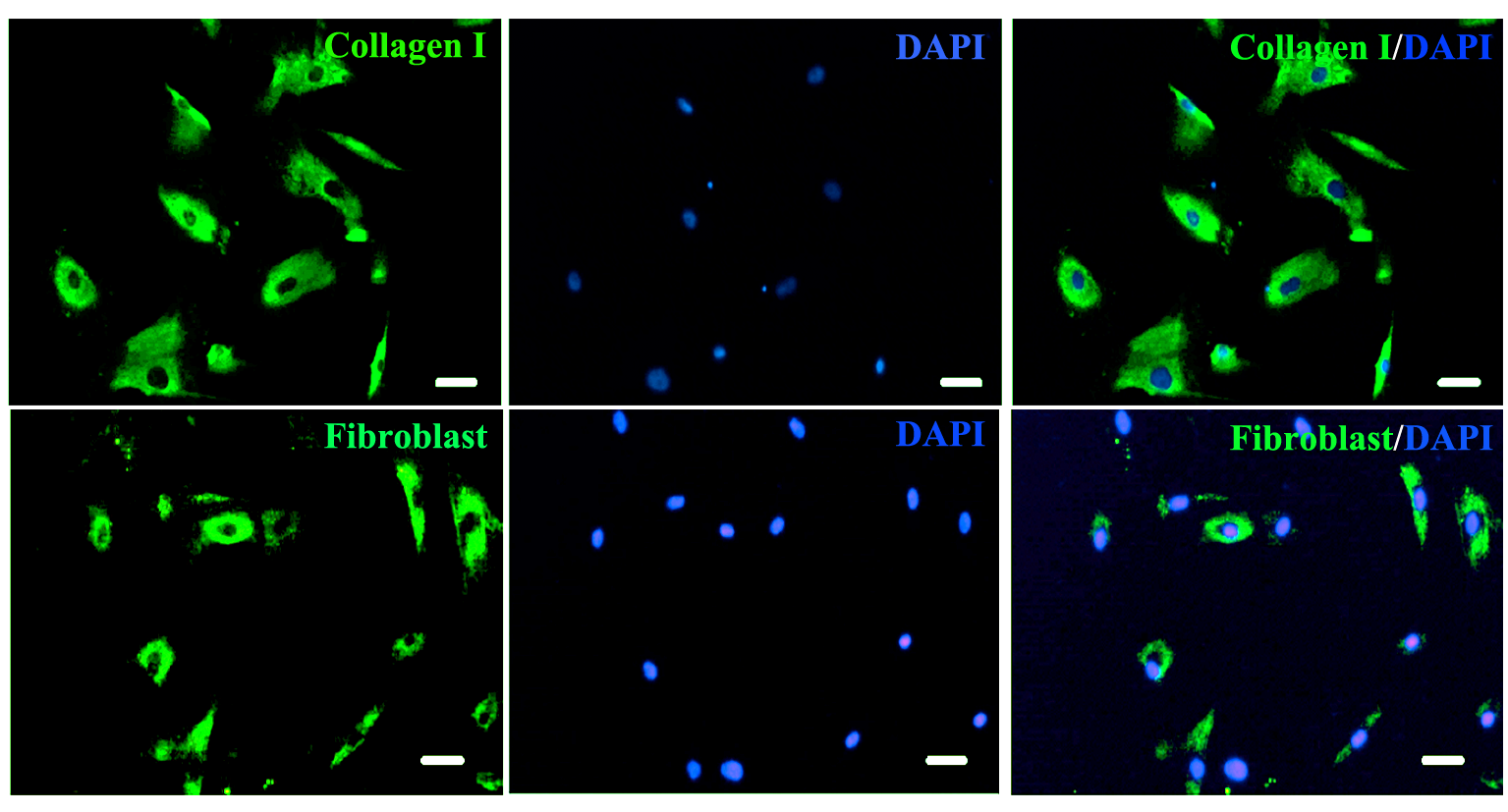

Supplement: Figure S1 — Immunocytochemical staining against anti-collagen I (top panel) and anti-human fibroblast antibodies in human atrial fibroblasts. Scale bar: 25 μm [file jcmm0017-1345-SD1.tif]

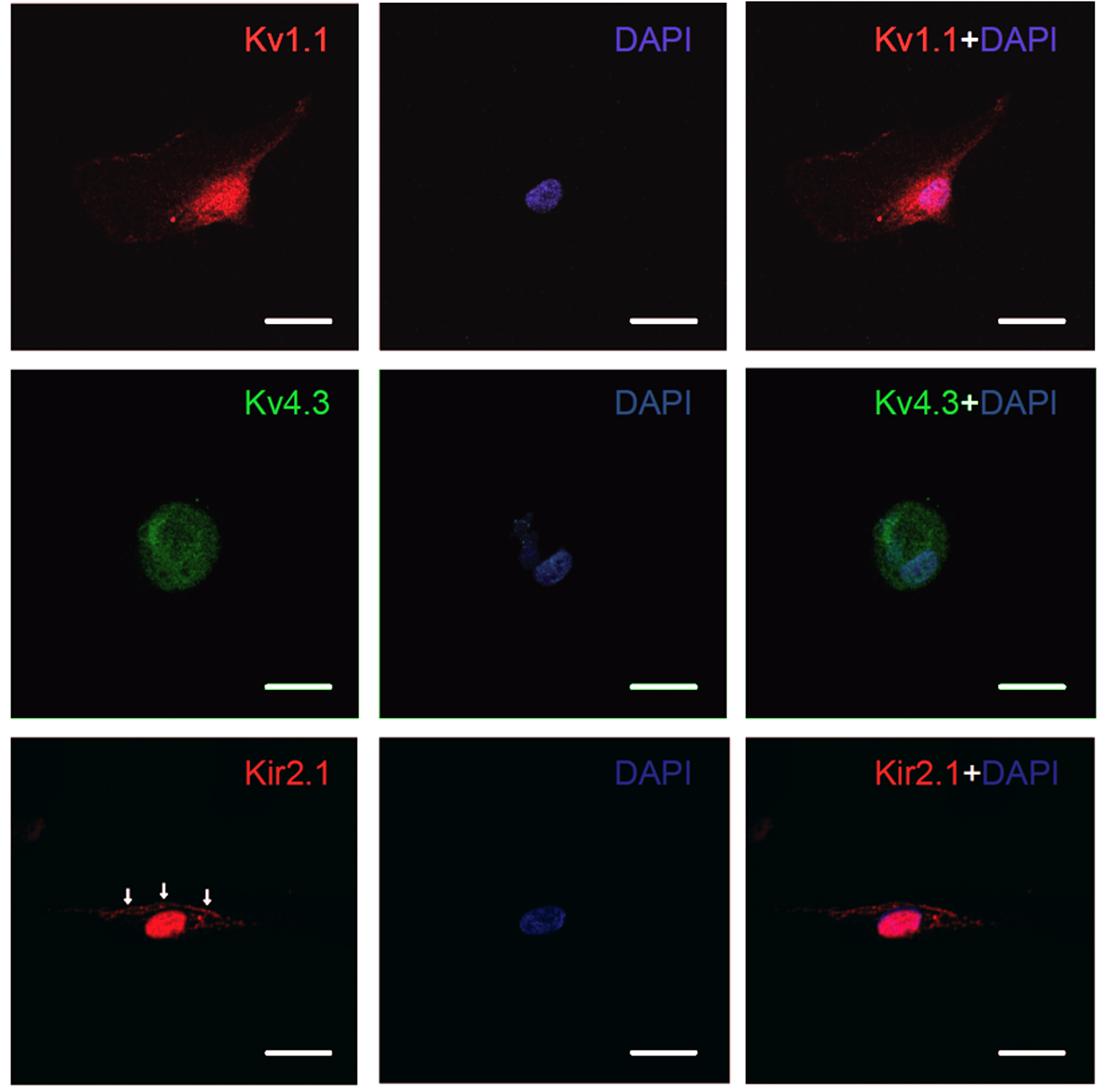

Supplement: Figure S2 — Immunocytochemical staining of Bkca (Kv1.1), Ito (Kv4.3) and IKir (Kir2.1) channels in human atrial fibroblasts. Scale bar: 50 μm. [file jcmm0017-1345-SD2.tif]
